# Copper Tolerance and Biosorption of *Saccharomyces cerevisiae* during Alcoholic Fermentation

**DOI:** 10.1371/journal.pone.0128611

**Published:** 2015-06-01

**Authors:** Xiang-yu Sun, Yu Zhao, Ling-ling Liu, Bo Jia, Fang Zhao, Wei-dong Huang, Ji-cheng Zhan

**Affiliations:** 1 College of Food Science and Nutritional Engineering, China Agricultural University, Beijing, 100083, P.R. China; 2 Faculty of Science, University of Copenhagen, København S, Denmark; University of Leicester, UNITED KINGDOM

## Abstract

At high levels, copper in grape mash can inhibit yeast activity and cause stuck fermentations. Wine yeast has limited tolerance of copper and can reduce copper levels in wine during fermentation. This study aimed to understand copper tolerance of wine yeast and establish the mechanism by which yeast decreases copper in the must during fermentation. Three strains of *Saccharomyces cerevisiae* (lab selected strain BH8 and industrial strains AWRI R2 and Freddo) and a simple model fermentation system containing 0 to 1.50 mM Cu^2+^ were used. ICP-AES determined Cu ion concentration in the must decreasing differently by strains and initial copper levels during fermentation. Fermentation performance was heavily inhibited under copper stress, paralleled a decrease in viable cell numbers. Strain BH8 showed higher copper-tolerance than strain AWRI R2 and higher adsorption than Freddo. Yeast cell surface depression and intracellular structure deformation after copper treatment were observed by scanning electron microscopy and transmission electron microscopy; electronic differential system detected higher surface Cu and no intracellular Cu on 1.50 mM copper treated yeast cells. It is most probably that surface adsorption dominated the biosorption process of Cu^2+^ for strain BH8, with saturation being accomplished in 24 h. This study demonstrated that *Saccharomyces cerevisiae* strain BH8 has good tolerance and adsorption of Cu, and reduces Cu^2+^ concentrations during fermentation in simple model system mainly through surface adsorption. The results indicate that the strain selected from China’s stress-tolerant wine grape is copper tolerant and can reduce copper in must when fermenting in a copper rich simple model system, and provided information for studies on mechanisms of heavy metal stress.

## Introduction

Copper (Cu) is unavoidable in winemaking: long-term use of copper fungicide [1–2] may increase the copper level in soil [3–4] and grape berry; winemaking equipment [5] and copper sulphate or copper citrate addition for eliminating H_2_S [6–7] may also increase the copper content in must. In a narrow range of low concentration, copper is an essential trace element in almost all organisms and plays an important positive role for organisms [8–10]. However, it would have inhibitory effect on cell when out of the useful range, even toxicity. A high level of Cu^2+^ such as 0.1 mM [11] in must inhibits yeast growth and activity; and high level Cu^2+^ is generally believed to cause sluggish fermentation (32 mg/l and 64 mg/l) [12] and a reduction in alcohol production (10.24 mg/l and 80.64 mg/l) [13]. At the same time, with the increase of copper content in wines, particularly existence with other heavy metals such as iron, manganese, zinc, nickel, lead, scandium etc, will cause harm to the health of consumers [14]. Maximum residue levels (MSL) of copper in European regulation is 20 mg/kg in grape must and 1 mg/L in wine [7]; China’s national regulation of wine, GB15037-2006, claims the same Cu MSL in wine but no limits in grape must.

In order to remove the excessive copper ions in wines, the current method is to add the adsorbent such as glue and then remove it by filtering. OIV allowed to add potassium ferrocyanide, bentonite, gum Arabic, polyvinylimidazole polyvinylpyrrolidone copolymers, chitin, chitosan et al. in wines to reduce the copper content, but these additives will affect wine sensory quality in different degrees, even detrimental to drinkers’ health [15–17]. And *Saccharomyces cerevisiae* has the capability of adsorption of copper ions [15–17]. Utilization *Saccharomyces cerevisiae* to complete the alcoholic fermentation and remove the redundant copper ions at the same time can not only ensure the safety of the quality of the wine, but also highly retain the original color and flavor of wine. Also it conforms to the requirements of the organic wine production, so it is a kind of environmental protection and effective method. Both non-living and living wine yeast were proved to have copper-uptake capacity of 0.04 to 0.2 mM Cu/g cell in solutions containing 6.4 to 256 mg/L copper [18–19]. But its mechanism is still under discussion. A major theory is biosorption with two phases: passive extracellular combination and active intracellular transportation [20–21]; in aqueous solution, the former phase was found to happen in the first 10 min and the latter reacted slowly and did not occur when the ratio of Cu^2+^ to biomass was below 100 nmol/mg [22], indicating two mutually independent phases. Ion exchange at plasma membrane and vacuolar accumulation were believed to be potentially important mechanisms for heavy metal tolerance [23]. But no significant difference was found between normal yeast and yeast with defective vacuoles in Cu^2+^ adsorption [24].

Since in a short period of time, Bordeaux mixture pesticides are still difficult to be replaced [25], and some vineyard soils in Germany [2] and Czech [3], and grapes and wines in Italy [7–8] were found exceeded Cu MSL of local regulation. In future, we will probably need to face the wine fermentation under high copper concentration and the extra problem to reduce the copper concentration of wines. With different yeast strains, the copper resistances and the copper adsorption capacity are different [26–27]. In this situation, if we could find some copper resistance strains and figure out the adsorption mechanism of *Saccharomyces cerevisiae*, it would be very helpful to face this problem.

In this research, we chose two industrial *Saccharomyces cerevisiae* strains AWRI R2 and Freddo which were commonly used by Chinese winemakers for its good fermentation performances, and one laboratory *Saccharomyces cerevisiae* strain BH8 which showed a good property to resist under many different stresses [25, 28–29], by analyzing copper concentration in must during fermentation and copper’s effect on their growth, fermentation performance, ultrastructure changes, and elemental analysis to study their copper tolerance and interaction with copper. Cu was added into model synthetic medium (MSM) in reference to fermentation tests in YNB medium and white grape must [12], reaching initial concentrations of 0.50, 1.00 and 1.50 mM, which would not be permitted in wine; the levels were only for research to hopefully demonstrate more clearly the absorption mechanism. The results could be used for selecting wine yeast strains with high copper-adsorption and resistance to high level copper in grape must during alcoholic fermentation, and could give a better understanding on the adsorption mechanism of *Saccharomyces cerevisiae* to copper.

## Materials and Methods

### Yeast strains

Three *Saccharomyces cerevisiae* strains were used; one laboratory strain, BH8 (B), separated (from BeiHong grape must) and stored at the laboratory (China Agricultural University, Beijing), identified as *S*. *cerevisiae* by Institute of Microbiology, Chinese Academy of Sciences [29]; two industrial strains, AWRI R2 (A; Maurivin Co., Australia) and Freddo (F; Erbslöh Co., Germany), commonly used by Chinese winemakers for their good fermentation performances.

Yeasts maintained on slants were pre-cultured aerobically to 6×10^7^ cfu/mL in shaking flasks containing 60 mL YPD medium (1% yeast extract, 2% peptone, and 2% glucose) at 28°C, 120 r/m [29].

### Medium

Model synthetic medium (MSM) simulating components of standard grape juice [30] was applied in studying fermentation characteristics of wine yeast, containing the following components (g/L): glucose (100), fructose (100), tartaric acid (3), citric acid (0.3), l-malic acid (0.3), MgSO4 (0.2), KH_2_PO_4_ (2). Nitrogen was adjusted to 190 mg total N/L with (NH_4_)_2_SO_4_ (0.3 g/L) and asparagine (0.6 g/L). Mineral salts (mg/L): MnSO_4_ H_2_O (4), ZnSO_4_ 7H_2_O (4), KI (1), CoCl_2_ 6H_2_O (0.4), (NH_4_)_6_Mo_7_O_24_ 4H_2_O (1), H_3_BO_3_ (1). Vitamins (mg/L): eso-inositol (300), biotin (0.04), thiamin (1), pyridoxine (1), nicotinic acid (1), pantothenic acid (1), p-amino benzoic acid (1). Fatty acids (mg/L): palmitic acid (1), palmitoleic acid (0.2), stearic acid (3), oleic acid (0.5), linoleic acid (0.5), linoleic acid (0.2).

### Fermentation experiments

CuSO_4_ 5H_2_O was added into MSM in a graded Cu^2+^ series of 0 (control), 0.50 mM (32 mg/L), 1.00 mM (64 mg/L) and 1.50 mM (96 mg/L) [12]. 4 mL yeast precultures were inoculated in 500 mL flasks containing 400 mL MSM to obtain a density of 10^6^ cells /mL [29]. Flasks were sealed with glass capillary stoppers filled with concentrated H_2_SO_4_ to prevent weight loss caused by water evaporation_._ Cultures were constantly shaken at 28°C, 120 r/m in thermostatic shaker (SKY-2102C, Shsukun Co. Ltd., Shanghai) [29]. Mass loss caused by CO_2_ evolution was monitored by weighing the fermentation flasks every 24 h [26]. Fermentation was considered to have stopped when mass loss was less than 0.02 g for 3 days. Samples of fermentation must were taken before and every 24 h after inoculation. Fermentation experiments were separated into two groups: one group for weighing, another group for sampling, and each group was carried out in triplicate.

### Determination of cell growth and viability

Cell growth was followed by measuring OD_600_ of the fermenting MSM [29] with a UV1800 spectrophotometer (Shimadzu, Japan). MSM free of Cu^2+^ was used as blank control. Viable cell level of strain B was determined by cell counting using the following procedure: 1 μL of five times diluted MSM sample was embedded on a cytometer and dyed with 1μL of 0.1% methylene blue, a dye commonly used in distinguishing viable and dead cells as it only stains dead cells. Total and viable cell were counted using optical microscope (COIC XSZ-3G) with 40× object lens. Survival rate was calculated following the equation viability % = V/T (V: viable cell amount; T: total cell amount).

### Analysis of fermentation performance

The remaining reducing sugars and ethanol content in samples taken during alcoholic fermentation were determined by HPLC using Waters 2414 RI Detector and BIO-RAD Aminex HPX-87H resin-based column (300*7.8mm) [31], which was eluted with 5 mM H_2_SO_4_ at 65°C, 0.6 mL/ min. Statistical differences for cell growth and fermentation performance of the strains were analyzed using single variable general liner model with PASW Statistics 18.

### Analysis of Cu biosorption

Cu adsorption of wine yeast strains were determined by measuring remaining copper in their fermenting MSM. A series of sterile MSM with 0.50, 1.00 and 1.50 mM Cu^2+^ were used as blank control. Samples taken during fermentation were filtrated by 0.45-μm cellulose acetate membrane filters; 4 mL of filtrate was dried at 105°C in 50 mL conical flask in dust-free drying oven, then digested with 5 mL HNO_3_-HClO_4_ (4:1, GR) adding in the flask which was then covered with watch glass, and heated on hot plate at 80°C for 2 h, then 120°C for 2 h, and 190°C until no white fog visible in the flask and the remaining liquid being clear and colorless; digested samples were washed by 18.2 MΩ ultrapure water and filtrated to 25 mL. Glassware were soaked overnight in 20% HNO_3_ and washed with ultrapure water before used. Cu concentrations of pre-treated samples were determined by ICP-AES (Perkin Elmer Optima 2000DV) at 327.393 nm. Removal ratio η and adsorption efficiency *A* (mg/g) of copper ion on yeast were calculated according to equations: η = (C_0_-C_1_)/C_0_, and *A* = (C_0_-C_1_) ×*V*/M, where C_0_ and C_1_ are initial and final Cu concentrations of MSM ferment, respectively, and *V* represents volume of sample, M means dry weight of yeast separated by centrifuge from the sample

### Structural analysis of yeast cell

Yeast cells for SEM-EDS and TEM-EDS were harvested by centrifugation of 10 mL of MSM sample at 4000 rpm (4°C, 10 min).

### SEM-EDS

Scanning electron microscope (SEM) was used to analyze the extracellular structure; and energy dispersive spectrometer (EDS) was used for surface elemental composition analysis. Harvested yeast cells were washed in deionized water three times by centrifugation and re-suspension. The cells were fixed with 2.5% glutaraldehyde-PBS overnight, washed in 30% PBS buffer (0.1 M, pH7.2) for three times (20 min each time), then post-fixed with 1% osmic acid (1 h), and washed three times with PBS. They were then dehydrated using ethanol with increasing concentrations (v/v) (30%, 50%, 70%, 80%, 90%, and 100%, each for three times, 20 min each time, followed by isoamyl acetate exchanging (three times, 20 min each time), critical point drying and gold crystal spraying. Pretreated cell samples were examined with SEM (Hitachi S-3400N) and SEM-EDS (Jeol JSM-6510A) [32].

### TEM-EDS

Intracellular structure was assessed by transmission electron microscopy (TEM) and EDS was combined with TEM for the elemental composition analysis of the cells. Harvested cells were fixed with 2% KMnO_4_ (4°C, overnight), washed in deionized water (six times, 15 min each time), dehydrated in a graded ethanol series (50%, 70%, 80%, and 90% for once, 100% for three times, 10 min once), then exchanged in ethanol-propanone (1:1, 8 min) and in anhydrous propanone (5 min), followed with a propanone-Epon812 mixture macerations (3:1, 1 h; 1:3, overnight; 1:1, 1 h; pure resin, 24 h), then embedded in Epon812 (37°C, 12 h). Pellets were polymerized by heating in an oven (60°C, 36 h), cut into 70 nm slices on an ultramicrotome (Leica EM UC6), double-dyed in uranyl acetate and lead citrate, and then examined with TEM (Hitachi H-7650) and TEM-EDS (FEI Tecnai F20) [33].

## Results

### Cell growth

**Fig 1:**
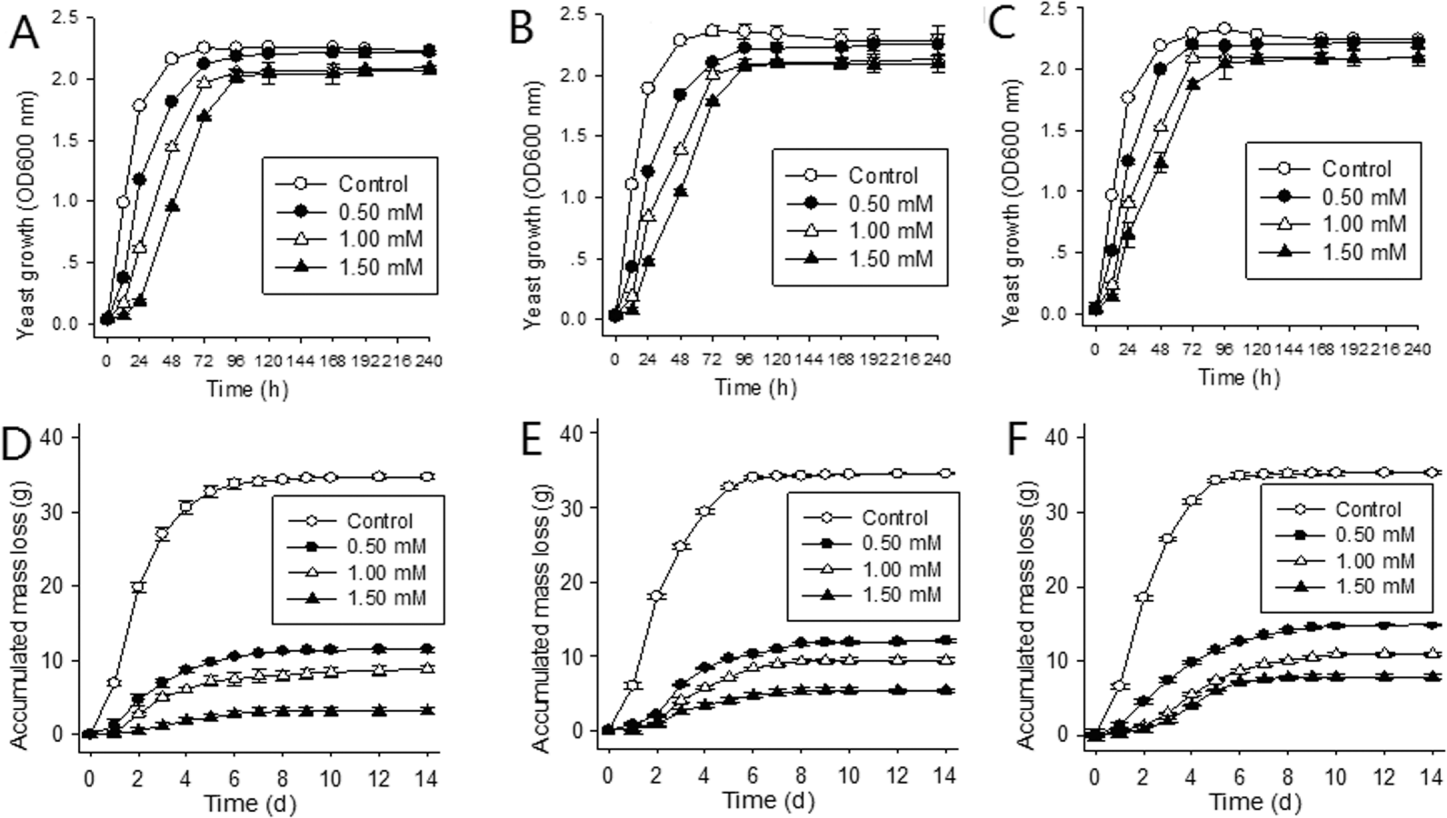
Growth curves (A, strain AWRI R2 (A); B, strain BH8 (B); C, strain Freddo (F)) and accumulated fermentation system mass loss(D, strain A; E, strain B; F, strain F) of *S*. *cerevisiae* strains during fermentation in MSM with 0 (control) (A*, B*, F*), 0.50 (A*, B**, F***), 1.00 (A*, B**, F***) and 1.50 mM (A*, B**, F***) Cu^2+^. (*, **, and *** represents different statistical significance level, CI = 0.95, n = 3).

Growth of all three yeast strains weakened as copper concentration increased (**Fig 1A, 1B and 1C**). Their growth in the control MSM was fastest, reaching log-phase and the end of growth after approximately 6 h and 48 h respectively, with maximum OD_600_ reaching about 2.25. For the 0.50 mM Cu^2+^ medium, the growth curve of strain F mimicked control but was delayed by about 12 hours; strains A and B were slower to start and the log-growth phase tapered off sooner than their controls although OD_600_ ultimately reached same levels as control. As copper concentration increased, all three strains were increasingly sluggish in growth with longer lag-phases and extended log-phases with lower growth rates, reaching stable-phase after 72 h with biomass (OD_600_ ratio) of approximately 80% of control. Copper was clearly inhibiting yeast growth. These results demonstrate that strain F had the highest growth and was the earliest one reached stable-phase, indicating a greater copper tolerance. Of the three strains strain B is more copper-tolerant than strain A and less than strain F.

### Fermentation performance

**Fig 2:**
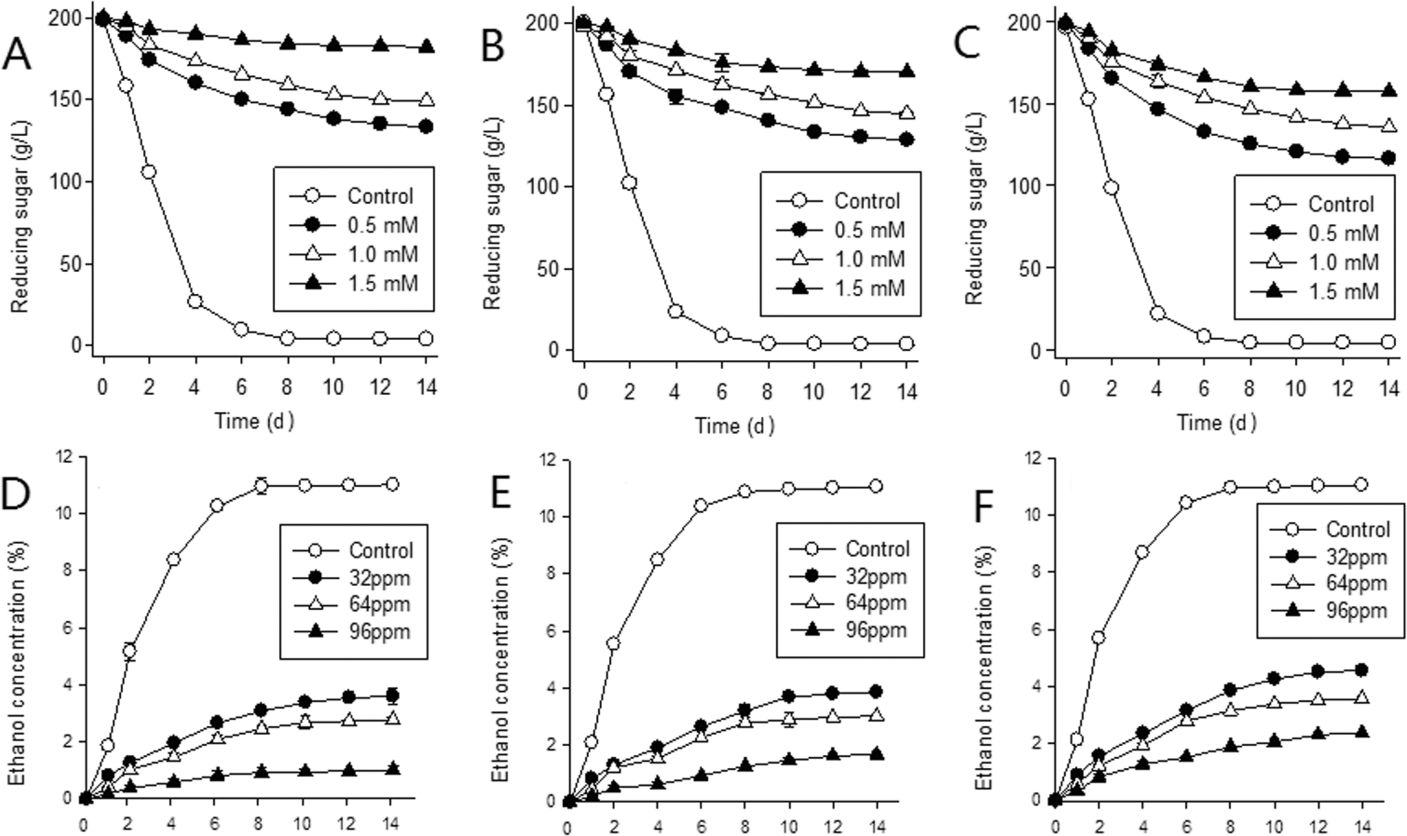
Fermentation must reducing sugar (A, strain A; B, strain B; C, strain F) and fermentation ethanol concentration (D, strain A; E, strain B; F, strain F) for *S. cerevisiae* strains in MSM with 0 (control) (A*, B*, F*), 0.50 (A*, B**, F***), 1.00 (A*, B**, F***) and 1.50 mM (A*, B**, F***) Cu^2+^. (*, **, and *** represent different statistical significance level, CI = 0.95, n = 3).

For all three strains the control ferments progressed most rapidly (**Fig 1D, 1E 1F and Fig 2**) in the first four days and remained stable after nine days. Total CO_2_ evolution was approximately 35g; residual reducing sugars were approximately 4g/L and ethanol concentration approximately 11% (v/v), with no statistical difference (CI = 0.95), indicating good fermentation performance by all strains. However in copper containing MSM, fermentations of all three strains were significantly affected, being sluggish or even becoming stuck, depending on Cu^2+^ concentration. In the 0.50 mM Cu^2+^ MSM, after 12 days total CO_2_ evolution of the three strains were less than 50% of their controls with statistically significant differences. Fermentation in 1.50 mM Cu^2+^ MSM lengthened to 14 days with total CO_2_ evolution being less than 23% of controls (9.08% (A), 15.59% (B) and 22.25% (F)). Corresponding to the growth curve (**Fig 1A, 1B and 1C**), the curves for the reducing sugars (**Fig 2A, 2B and 2C**) and ethanol (**Fig 2D, 2E and 2F**) became stable earlier and changed less with increasing copper concentration.

For the corresponding concentrations of Cu^2+^, growth activity and fermentation efficiency (**Figs 1 and 2**) of strain F was the highest, followed by strain B with strain A being the lowest (such as under 0.5 mM, at 24h, the OD value of strain A was 1.171, strain B 1.208, strain F 1.245; the alcohol production of strain A was 0.81, strain B 0.82, strain F 0.88)

### Copper biosorption

**Fig 3:**
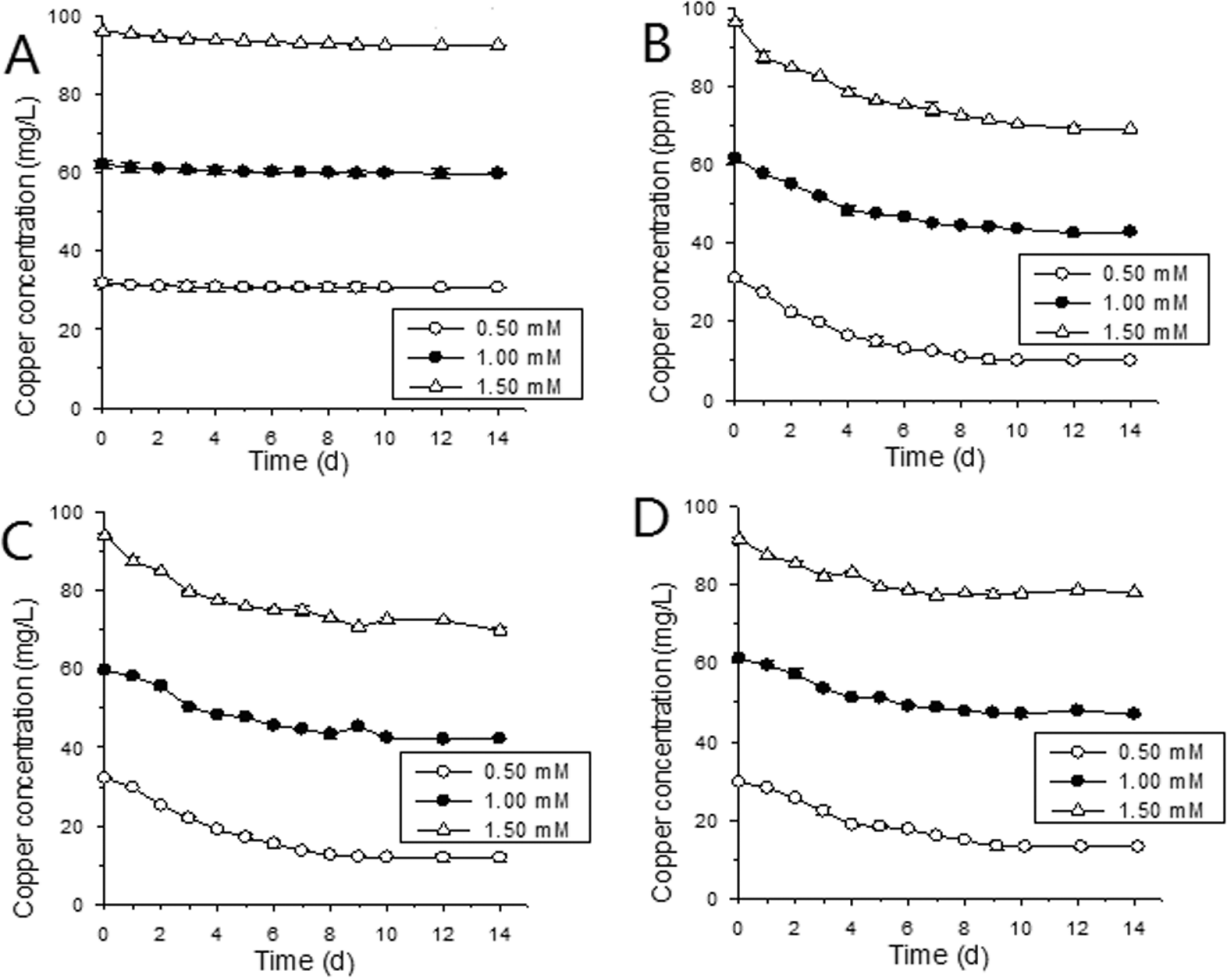
Copper ion concentration (A, Control; B, strain A; C, strain B; D, strain F) of MSM during fermentation for *S*. *cerevisiae* strains in MSM with 0 (control) (A*, B*, F*), 0.50 (A*, B**, F***), 1.00 (A*, B**, F***) and 1.50 mM (A*, B**, F***) Cu^2+^. (*, **, and *** represent different statistical significance level, CI = 0.95, n = 3).

**Fig 4:**
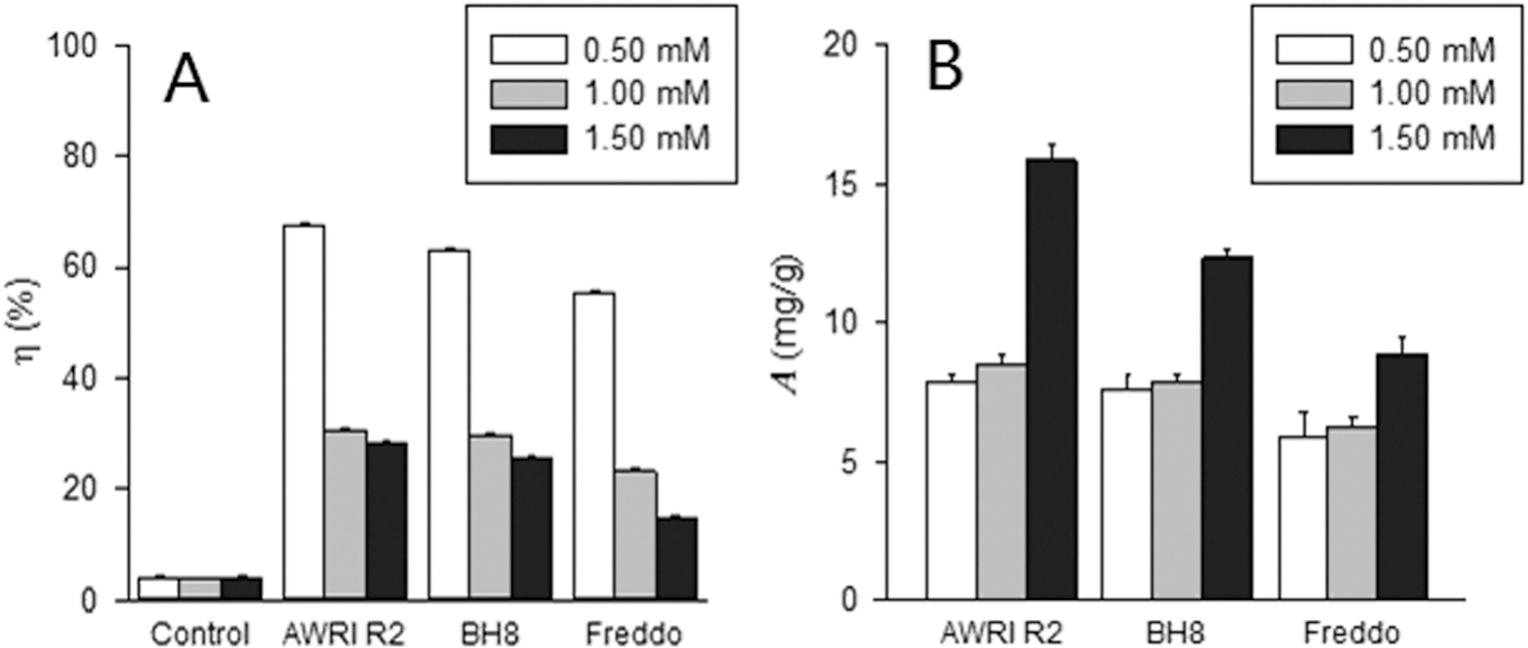
Removal ratio η (A) and adsorption efficiency *A* (B) of Cu^2+^ on *S*. *cerevisiae* strains AWRI R2 (A), BH8 (B) and Freddo (F) at the end of alcoholic fermentation in MSM with 0.50, 1.00 and 1.50 mM Cu^2+^.

It can be seen from **Fig 3**that Cu ion concentration of MSM ferments for all three strains went down with time during fermentation, with first four days decreasing rapidly and later period slowly (**Fig 3**), corresponding to the fermentation performance (**Fig 1D, 1E 1F and Fig 2**), indicating relations between yeast activity and copper biosorption. Contrarily, Cu ion concentrations in control with no yeast did not reduce significantly (**Fig 3**). Higher initial copper concentration correlated with lower removal ratio and higher adsorption efficiency; this can been seen from a significant drop of Cu removal ratio (**Fig 4A**) between groups of 0.50 mM and 1.00 mM initial Cu^2+^ and a leap upward of Cu biosorption by unit yeast (**Fig 4B**) between groups of 1.00 mM and 1.50 mM initial Cu^2+^ for all three yeast strains. The increase of adsorption efficiency as initial Cu concentration rises could be explained by yeast biomass decrease. Compared with strain A and F, strain B showed a medium removal ratio and adsorption efficiency under all three initial Cu levels (0.50, 1.00 and 1.50 mM; **Fig 4**). Among three yeast strains, strain A showed the strongest removal ratio (67.37%, **Fig 4A**) in 0.50 mM Cu and highest adsorption efficiency (15.82 mg/g, **Fig 4B**) in 1.50 mM Cu.

### Impacts of Cu on survival rate

To directly reflect copper’s lethal effect and the copper tolerance of strain B, living and dead yeasts were counted separately to calculate survival rates at different Cu^2+^ concentrations. The survival rate of strain BH8 (**Table 1**) increased with time in 24 h at each Cu^2+^ level, and decreased as Cu^2+^ concentration increased, indicating a lower reproduction capacity under the stress of high levels of Cu^2+^.

**Table 1:** Viability of *S*. *cerevisiae* BH8 at 12 h and 24 h in MSM with 0, 0.5, 1.0 and 1.5 mM Cu^2+^.

Cu^2+^ level (mM)	viability %	
	12 h	24 h
0.00	96.48±0.47^a^	99.97±0.86 ^a^
0.50	36.08±0.21 ^a^	66.48±0.39 ^a^
1.00	16.21±0.06 ^a^	23.47±0.45 ^a^
1.50	8.58±0.15 ^a^	11.13±0.09 ^a^

a. Mean value and SD for three independent fermentations.

### Impacts of Cu on surface morphology and element

**Fig 5:**
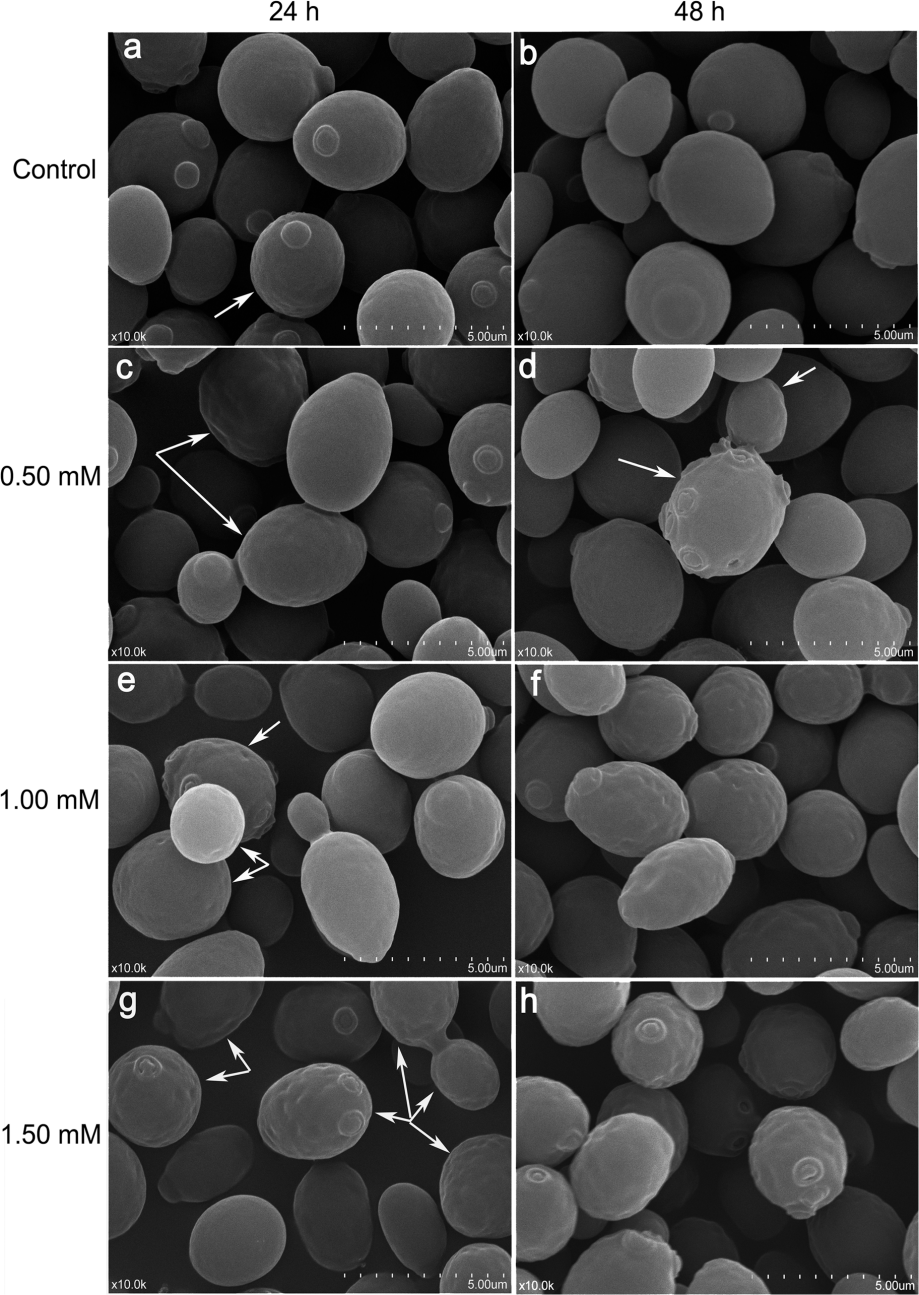
Images (×10000) of yeast surface of *S. cerevisiae* strain BH8 cultivated in MSM with 0 (control), 0.50, 1.00 and 1.50 mM Cu^2+^ for 24 h and 48 h. Arrows indicate pits on individual cell surfaces.

**Fig 6:**
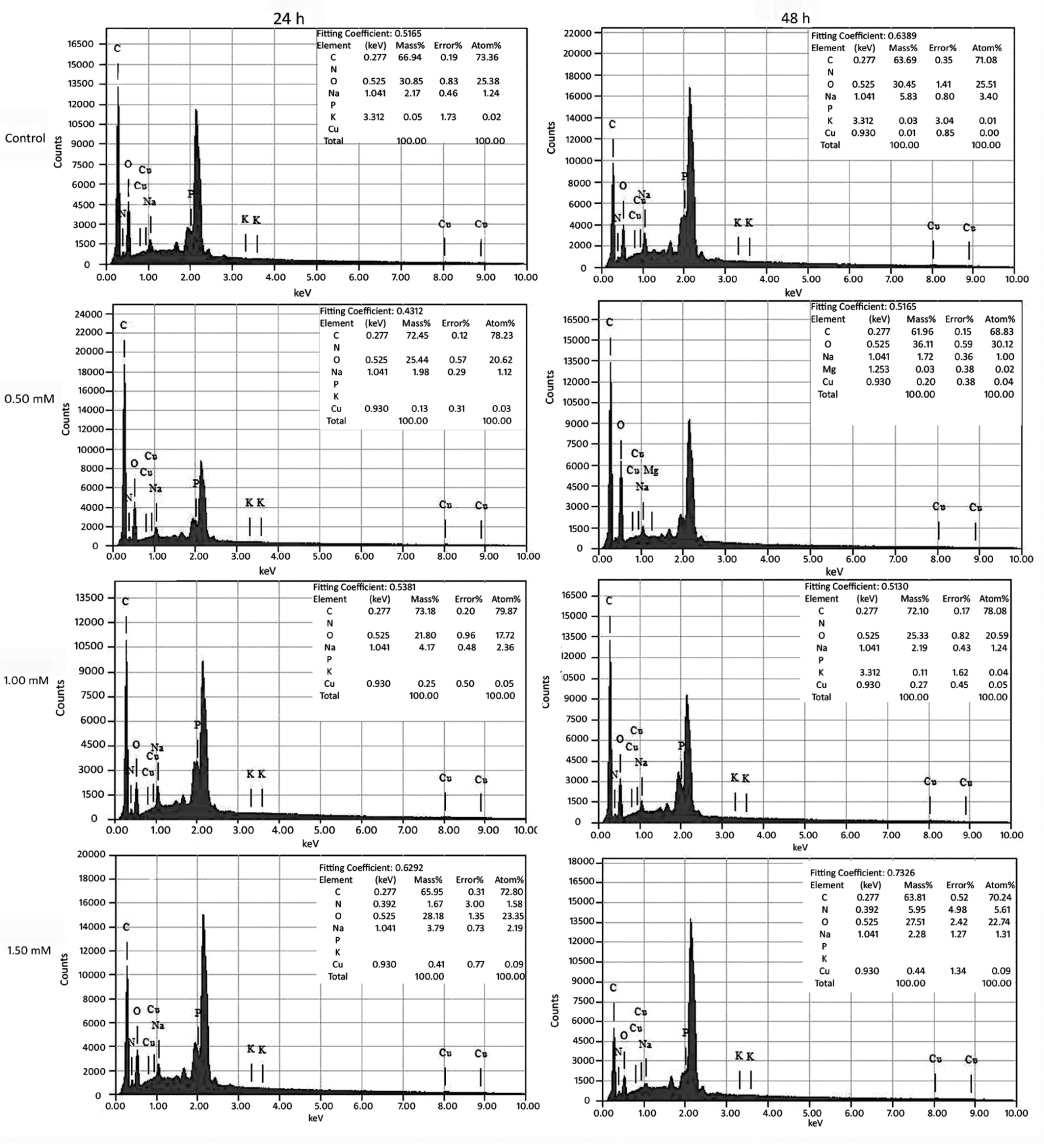
Elemental composition of yeast surface of *S. cerevisiae* strain BH8 cultivated in MSM with 0 (control), 0.50, 1.00 and 1.50 mM Cu^2+^ for 24 h and 48 h.

SEM images (**Fig 5**) show that toxic effects of Cu^2+^ on strain B lead to increasing changes in micromorphology with increasing time and Cu^2+^ concentration. EDS results (**Fig 6**) indicated cell surface of strain B mainly consists of carbon (C; over 60%) and oxygen (O; over 20%), with the mass fraction (Mass %) and atom% (at %) of Na decreasing with Cu increasing with time after Cu treatment. Gold (peak at 2.00 to 3.00 keV) parameters were not calculated with EDS since gold was sprayed on cell surface during the preparation for SEM testing. As the SEM images showed, yeast cells for the controls (**Fig 5A and 5B**) were orbicular-ovate, 4 to 6 μm long and 2 to 4 μm wide with smooth surfaces and no intercellular adhesions; besides that, there were also a little fold occurred on some individual cells, which could be a natural consequence of the sample preparation (centrifugal, deionized water washing, and ethanol dehydration) [34]; what’s more, buds and bud scars were no more than three for per cell. EDS didn’t detected Cu peak (**Fig 6**). In 0.50 mM Cu^2+^ treatment level, most cells remained regular oval at 24 h (**Fig 5C**), but cell deformation and pitted surface became obvious and occurred on more cells; after 48 h, there were more bud scars for per cell (**Fig 5D**). Meanwhile, potassium (K) was undetectable with EDS while Cu was detected with atom% less than 0.05% (**Fig 6**). At 1.00 mM Cu^2+^, the cells were slightly deformed and pitted after 24 h (**Fig 5E**) and significantly stretched with deep pits on most cells after 48 h (**Fig 5F**). For 1.00 mM Cu^2+^ treatment, the EDS results was resembles to those of 0.50 mM with no K peak; copper was slightly higher in mass% but still low as atom% of 0.05% (**Fig 6**). In 1.50 mM Cu^2+^ treatment, cells were mostly rough and significantly pitted on the surface with some being stretched with adhesion by 24 h (**Fig 5G**), and almost all yeast cells were deformed having significantly rough and uneven surfaces by 48 h (**Fig 5H**); nitrogen (N) was detected in increasing amounts with fermentation time and Cu was higher in the EDS results, K peak was also not detected (**Fig 6**). Base on these results, we deduced that the disappearance of K peak and decreasing of Na peak with increasing of Cu peak with time after Cu treatment might have certain relations.

### Impacts of Cu on intracellular morphology and element

**Fig 7:**
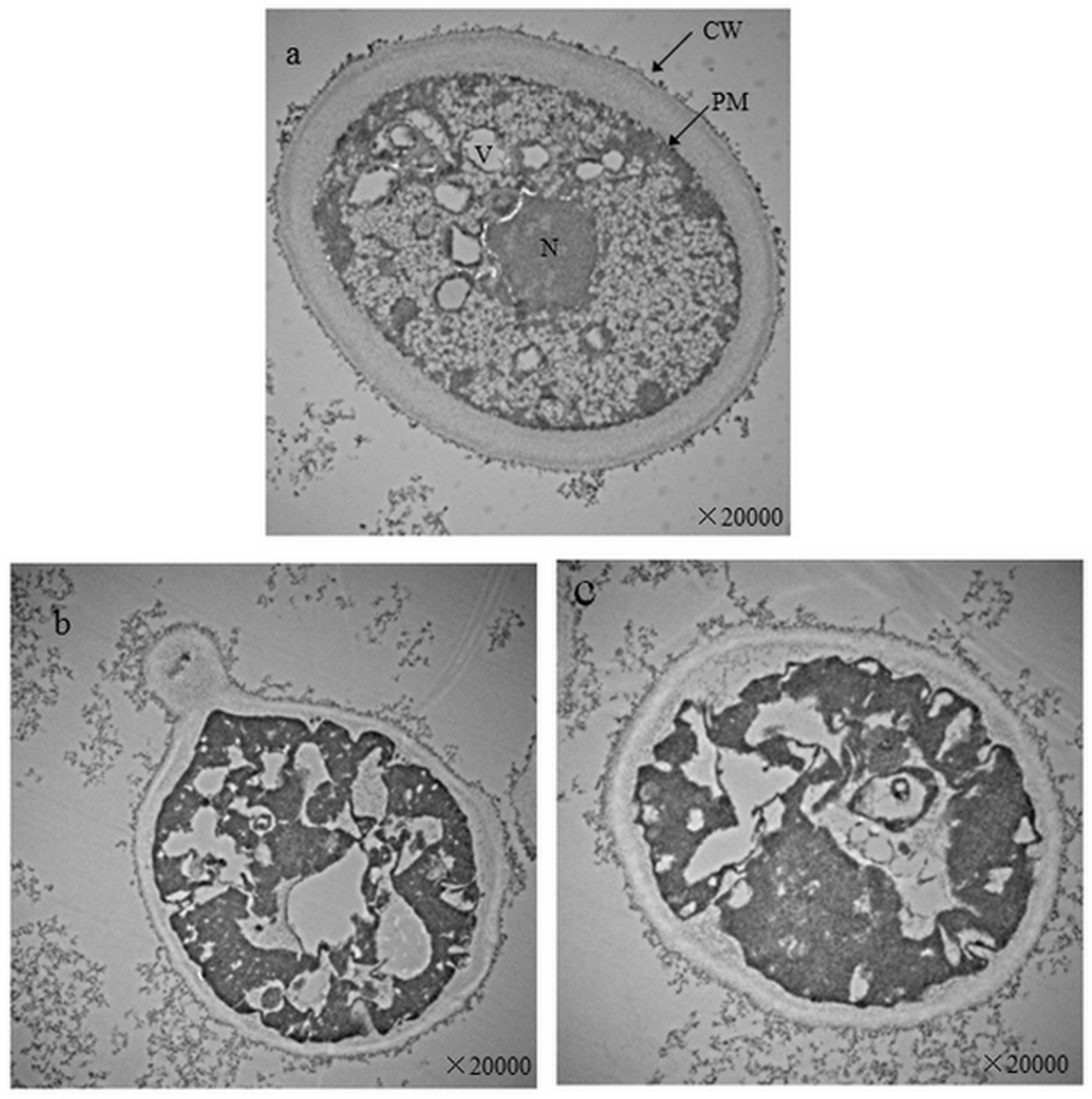
Intracellular images (×20000) of *S*. *cerevisiae* strain BH8 before (a) and after (b, c) culturing in MSM with 1.50 mM Cu^2+^ for 48 h; CW: cell wall; N: cell nuclear; PM: plasma membrane; V: vacuole.

**Fig 7**presents intracellular images (×20000) of strain B examined with TEM. The yeast from the control (**Fig 7A**) was a normal oval shape with complete cell walls and plasma membranes. The cell wall thickness was even, the organelles dispersed in plasma, and the vacuoles were small and of a similar size. In contrast the yeast taken after 48 h from the MSM ferment containing 1.50 mM Cu^2+^ (**Fig 7B**), had rough cell walls and plasma membranes which is in agreement with its SEM image (**Fig 5H**). Plasmolysis occurred with uneven plasma distribution and organelles could not be distinguished. This cytoplasm contraction could be related to Cu^2 +^ induced lipid peroxide activity in the plasma membrane.

**Fig 8:**
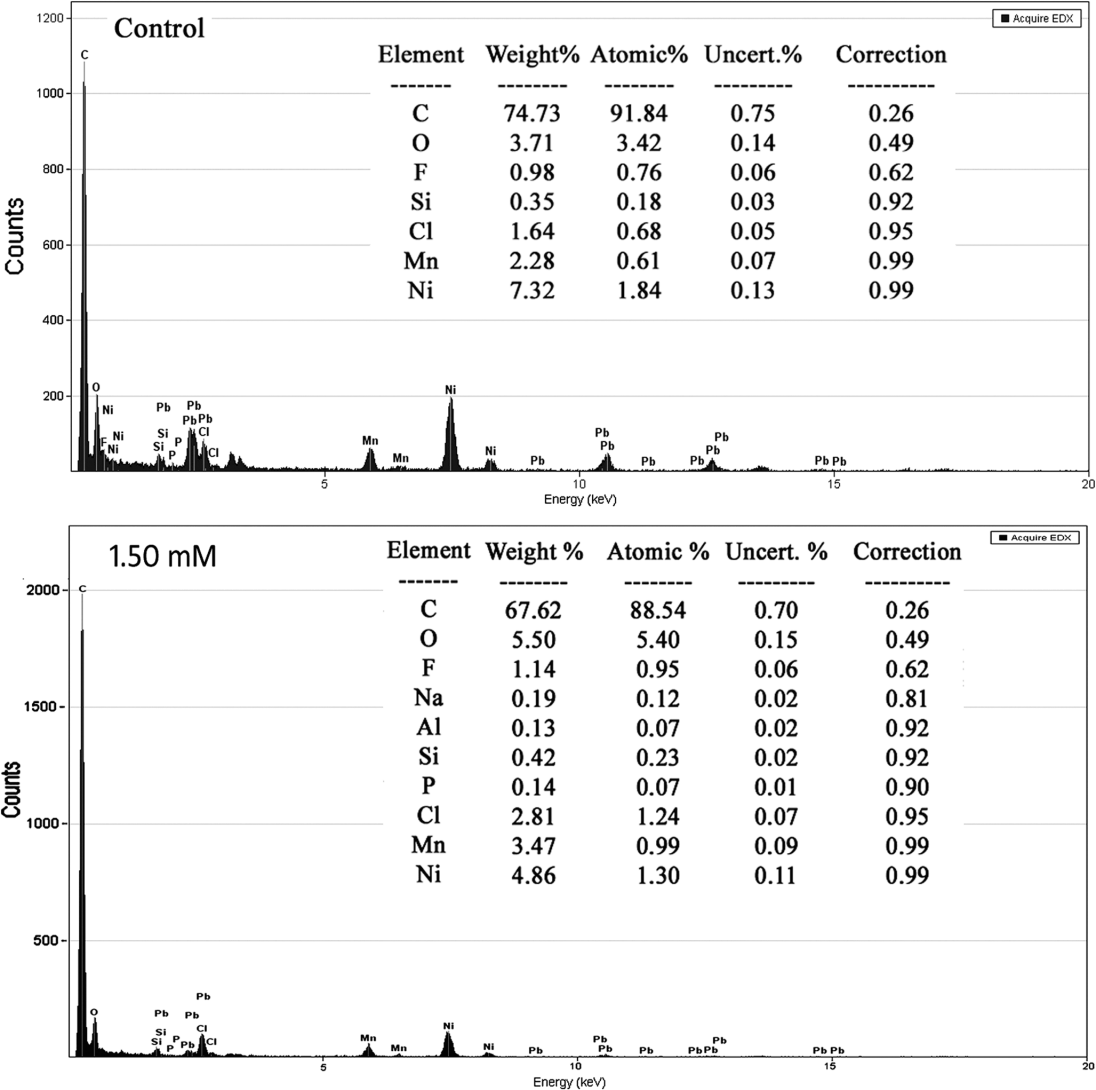
Intracellular elemental composition of *S*. *cerevisiae* BH8 before (a) and after (b) cultivated in MSM with 1.50 mM Cu^2+^ for 48h.

**Fig 8**shows the intracellular element ratios determined by TEM-EDS using the same *S*. *cerevisiae* BH8 sample of **Fig 7**. Lead (Pb) was introduced into yeast cell from the sample preparation. Yeast mainly consists of carbon and oxygen, which is in agreement with composition of yeast surface (**Fig 6**). Compared to the control, yeast in 1.50 mM Cu^2+^ for 48 h had no significant changes in C and O although there was some N present. No copper peak was detected, and the atomic ratio (Atomic %) of Ni decreased by 0.54 (**Fig 8**), indicating Ni^+^ leakage after cell membrane deformation. That no Cu was detected inside the yeast cells suggests that strain B does not accumulate Cu^2+^ in its cell and living cells of strain B reduce Cu^2+^ mainly by surface adsorption.

The mechanism of surface Cu^2+^ adsorption could be further studied with atomic force microscopy and confocal laser scanning microscopy. However the sample preparation method needs to be studied since the reported methods for other microorganisms have failed on *Saccharomyces cerevisiae*.

## Discussion

As there are many restrictions on natural grape juice, such as the supply of seasonal restrictions, the difference of grape juice composition caused by viticulture region and grape varieties, and the effect of solid composition of natural grape juice on separation of yeast cells, for a long time the studies on *Saccharomyces cerevisiae* with different researchers were hard to consistent. And MSM has the advantages of easy using, good reproducibility et al [30], in this study, we chose MSM rather than natural grape juice as fermentation medium.

At a low concentration range, copper is a necessary metal elements for biological growth and metabolism and cofactors for intracellular enzymes metabolism [27]. But once grossing over the beneficial range, it will have inhibitory effect on cells, even toxicity [35]. In wine making, high copper content also affects the wine fermentation process and wine quality. In this experiment, in the MSM medium without copper, all three strains showed a good growth activity and fermentation performance, and the growth curve, CO_2_ release quantity, reducing sugar utilization and alcohol production were similar between these three strains (**Figs 1 and 2**). Once copper was added into the MSM medium, the growth of *Saccharomyces cerevisiae* was delayed even stagnated, and the effect was positive correlated with the copper concentration. This was in agreement with the result of Shanmuganathan et al [36]. A possible reason for this could be that the yeast cell accumulates large amounts of reactive oxygen species (ROS) at the high concentrations, leading to protein denaturation, membrane order alteration and damage to intracellular enzyme and consequent reduced metabolism, and ultimately cell death [36]. Therefore, anaerobic fermentation was not possible with the reducing sugars not being able to be used for energy.

For the copper biosorption of *Saccharomyces cerevisiae*, in this experiment, even if they didn't add yeast, the copper concentration would reduce slightly with the extension of time **(Fig 3A)**. The possible reason was that copper and a small amount of sulfur ions in the solution formed precipitation [25]. After fermentation, the removal rate was 14.86~67.37% and the adsorption efficiency was 5.88~15.82 mg/g. This was in consistent with Volesky et al study [37] and Donmez et al study [38], in their study, the adsorption efficiency was 2~40 mg/g. With different yeast strains, the adsorption quantity of *Saccharomyces cerevisiae* was different. For these three strains, the highest removal rate and the highest adsorption efficiency were all strain A, while strain F was the lowest. In Brandolini et al study [26], the cell growth and fermentation performance of copper resistant yeast strain behaved better than normal yeast strain, and could absorb more copper ions too. By contrast, in this experiment, strain B behaved better on cell growth and fermentation performance under copper stress, but for the copper removal ratio, strain A was better. Liu [39] also reported similar results. This difference might be related with the tested strain features. Also, with different initial copper concentrations, the adsorption quantity of *Saccharomyces cerevisiae* was different. In this experiment, though with higher initial copper concentrations, the toxicity of copper on yeast was higher and leaded to yeast biomass decreased and the removal rate decreased, but the adsorption efficiency increased, which was in agreement with the previous research results [40].

In order to could give a better understanding on the adsorption mechanism of *Saccharomyces cerevisiae* to copper, SEM-OES and TEM-OES were used to observe the ultrastructure change and elemental transformation. In SEM-OES observation, yeast cell surfaces became uneven after copper adsorption together with potassium peak disappeared while copper and nitrogen appeared, but the contents of these elements were very low. It might be under coverage of the high gold peak value, or indicating a small capacity of surface copper adsorption by strain B. With the increasing of the initial copper concentration, the cell surface was more and more roughness, the copper content was getting higher and the K:Cu ratio continued decreased (**Fig 8**), indicated that the adsorption of copper might be associated with the release of potassium from the cell surface. When the copper concentration reached 1.50 mM, nitrogen peak appeared on cell surface, and with the extension of time, the nitrogen peak enhanced. This might because that the copper began to complex with Nitrogen groups of MSM. This was in agreement to Brady et al study [40]. They found 70% of K^+^ was rapidly released during Cu^2+^ biosorption in waste water. Hence, it might indicate that ion exchange was involved in the biosorption of Cu^2+^ during fermentation. With the extension of time, though the cell surface was more and more roughness, the copper content was basically remain unchanged, which means the copper adsorption quantity on the yeast surface had reached saturation at the point of 24 h or before. In previous study in waste water, the adsorption of *Saccharomyces cerevisiae* on copper was divided into two stages, the first phase happened quickly on cell surface without energy consumption and the second phase was a long and slow intracellular accumulation and transformation process involving metabolism [38, 40]. The SEM-OES results were fit with the first phase.

Then TEM-EDS was used to observed the intracellular structure and elemental transformation. Under copper stress, the thickness of *Saccharomyces cerevisiae* cell wall was not uniform, the cytoplasm shrank and uneven distribution, organelles couldn't be recognized. The reason of cytoplasm shrank might be related with the lipid over oxidation of cell plasma membrane. When the copper concentration reached 1.50 mM, nitrogen peak appeared on cell surface, the intracellular Potassium content reduced, further illustrated ion exchange was involved in the biosorption of Cu^2+^ during fermentation. And there was no copper detected in intracellular, indicated that *Saccharomyces cerevisiae* could not transport copper into internal. In combination with the results of SEM-OES, the main adsorption mechanism of *Saccharomyces cerevisiae* to copper during alcoholic fermentation was cell surface adsorption. As to whether intracellular accumulation exists, it still needs further studies to confirm.

In conclusion, copper stress could delay even stagnate the growth of *Saccharomyces cerevisiae*, reduce the reducing sugar uptake and ethanol production, and the degree was related to the initial copper concentration and strains. The copper tolerance and copper adsorption ability of strains showed a negative correlation. After *Saccharomyces cerevisiae* adsorbed copper, the yeast surface and intracellular all changed irregularly. Ion exchange was involved in the biosorption of Cu^2+^ during fermentation, and the main adsorption mechanism of *Saccharomyces cerevisiae* to copper during alcoholic fermentation was cell surface adsorption, reaching saturation in 24 h.

## Supporting Information

S1 Table Data for Fig 1A: growth curves of strain A. (DOC)

S2 Table Data for Fig 1B: growth curves of strain B. (DOC)

S3 Table Data for Fig 1C: growth curves of strain F. (DOC)

S4 Table Data for Fig 1D: accumulated fermentation system mass loss of strain A. (DOC)

S5 Table Data for Fig 1E: accumulated fermentation system mass loss of strain B. (DOC)

S6 Table Data for Fig 1F: accumulated fermentation system mass loss of strain F. (DOC)

S7 Table Data for Fig 2A: fermentation must reducing sugar of strain A. (DOC)

S8 Table Data for Fig 2B: fermentation must reducing sugar of strain B. (DOC)

S9 Table Data for Fig 2C: fermentation must reducing sugar of strain F. (DOC)

S10 Table Data for Fig 2D: fermentation ethanol concentration of strain A (DOC)

S11 Table Data for Fig 2E: fermentation ethanol concentration of strain B. (DOC)

S12 Table Data for Fig 2F: fermentation ethanol concentration of strain F. (DOC)

S13 Table Data for Fig 3A: copper ion concentration of MSM during fermentation for control group. (DOC)

S14 Table Data for Fig 3B: copper ion concentration of MSM during fermentation for strain A. (DOC)

S15 Table Data for Fig 3C: copper ion concentration of MSM during fermentation for strain B. (DOC)

S16 Table Data for Fig 3D: copper ion concentration of MSM during fermentation for strain F. (DOC)

S17 Table Data for Fig 4A: removal ratio ηof Cu^2+^ on *S*. *cerevisiae* strains AWRI R2 (A), BH8 (B) and Freddo (F) at the end of alcoholic fermentation in MSM with 0.50, 1.00 and 1.50 mM Cu^2+^. (DOC)

S18 Table Data for Fig 4B: adsorption efficiency *A* of Cu^2+^ on *S*. *cerevisiae* strains AWRI R2 (A), BH8 (B) and Freddo (F) at the end of alcoholic fermentation in MSM with 0.50, 1.00 and 1.50 mM Cu^2+^. (DOC)
