# Supplementary material for: Copper Tolerance and Biosorption of Saccharomyces cerevisiae during Alcoholic Fermentation
Source: PLoS One. 2015 Jun 1;10(6):e0128611. doi: 10.1371/journal.pone.0128611 (PMC4452488; doi:10.1371/journal.pone.0128611)
Supplement: S1 Table — (DOC) [file pone.0128611.s001.doc]

**S1 Table** Data for Fig 1 A: growth curves of strain A.

| fermentation time (h) | yeast growth (OD 600 nm) | | | |
| --- | --- | --- | --- | --- |
|  | 0 mM group | 0.5 mM group | 1 mM group | 1.5 mM group |
| 0 | 0.028±0.0075 | 0.033±0.002603 | 0.04±0.001528 | 0.044±0.0065 |
| 12 | 0.986±0.0045 | 0.369±0.013115 | 0.165±0.014193 | 0.061±0.016 |
| 24 | 1.777±0.012 | 1.172±0.008819 | 0.616±0.018339 | 0.18±0.019 |
| 48 | 2.161±0.0295 | 1.812±0.001528 | 1.438±0.022234 | 0.958±0.003 |
| 72 | 2.252±0.002 | 2.12±0.012991 | 1.964±0.023497 | 1.693±0.0105 |
| 96 | 2.25±0.005 | 2.184±0.015431 | 2.055±0.024835 | 2.016±0.046 |
| 120 | 2.26±0.012 | 2.205±0.016093 | 2.061±0.024265 | 2.045±0.085 |
| 168 | 2.254±0.007 | 2.216±0.012914 | 2.074±0.01837 | 2.04±0.083 |
| 192 | 2.244±0.004 | 2.212±0.033287 | 2.077±0.019519 | 2.059±0.016093 |
| 240 | 2.23±0.0015 | 2.218±0.013642 | 2.091±0.018889 | 2.07±0.012914 |
