# Supplementary material for: Copper Tolerance and Biosorption of Saccharomyces cerevisiae during Alcoholic Fermentation
Source: PLoS One. 2015 Jun 1;10(6):e0128611. doi: 10.1371/journal.pone.0128611 (PMC4452488; doi:10.1371/journal.pone.0128611)
Supplement: S2 Table — (DOC) [file pone.0128611.s002.doc]

**S2 Table** Data for Fig 1 B: growth curves of strain B.

| fermentation time (h) | yeast growth (OD 600 nm) | | | |
| --- | --- | --- | --- | --- |
|  | 0 mM group | 0.5 mM group | 1 mM group | 1.5 mM group |
| 0 | 0.023±0.01837 | 0.033±0.007 | 0.041±0.012914 | 0.047±0.023497 |
| 12 | 1.103±0.019519 | 0.429±0.004 | 0.189±0.033287 | 0.076±0.024835 |
| 24 | 1.888±0.018889 | 1.208±0.0015 | 0.839±0.013642 | 0.466±0.024265 |
| 48 | 2.281±0.025887 | 1.837±0.0065 | 1.391±0.01392 | 1.046±0.01837 |
| 72 | 2.357±0.03985 | 2.102±0.016 | 1.998±0.033451 | 1.7859±0.019519 |
| 96 | 2.354±0.059221 | 2.218±0.019 | 2.086±0.042226 | 2.071±0.018889 |
| 120 | 2.337±0.066484 | 2.2188±0.003 | 2.101±0.036191 | 2.089±0.025887 |
| 168 | 2.287±0.089414 | 2.226±0.0105 | 2.107±0.033118 | 2.089±0.03985 |
| 192 | 2.28±0.103233 | 2.249±0.046 | 2.122±0.023714 | 2.086±0.059221 |
| 240 | 2.285±0.124156 | 2.251±0.085 | 2.126±0.032002 | 2.093±0.066484 |
