# Supplementary material for: Copper Tolerance and Biosorption of Saccharomyces cerevisiae during Alcoholic Fermentation
Source: PLoS One. 2015 Jun 1;10(6):e0128611. doi: 10.1371/journal.pone.0128611 (PMC4452488; doi:10.1371/journal.pone.0128611)
Supplement: S3 Table — (DOC) [file pone.0128611.s003.doc]

**S3 Table** Data for Fig 1 C: growth curves of strain F.

| fermentation time (h) | yeast growth (OD 600 nm) | | | |
| --- | --- | --- | --- | --- |
|  | 0 mM group | 0.5 mM group | 1 mM group | 1.5 mM group |
| 0 | 0.034±0.002 | 0.037±0.012991 | 0.041±0.023497 | 0.045±0.046 |
| 12 | 0.968±0.005 | 0.513±0.015431 | 0.235±0.024835 | 0.138±0.023714 |
| 24 | 1.766±0.012 | 1.245±0.016093 | 0.904±0.024265 | 0.649±0.103233 |
| 48 | 2.192±0.007 | 1.998±0.012914 | 1.526±0.01837 | 1.236±0.085 |
| 72 | 2.29±0.004 | 2.201±0.033287 | 2.091±0.019519 | 1.869±0.032002 |
| 96 | 2.331±0.0015 | 2.191±0.013642 | 2.098±0.018889 | 2.049±0.124156 |
| 120 | 2.281±0.0065 | 2.203±0.01392 | 2.098±0.025887 | 2.073±0.019 |
| 168 | 2.247±0.016 | 2.213±0.033451 | 2.094±0.03985 | 2.083±0.042226 |
| 192 | 2.246±0.019 | 2.215±0.042226 | 2.088±0.059221 | 2.087±0.059221 |
| 240 | 2.242±0.003 | 2.215±0.036191 | 2.094±0.066484 | 2.097±0.018889 |
