# Supplementary material for: Copper Tolerance and Biosorption of Saccharomyces cerevisiae during Alcoholic Fermentation
Source: PLoS One. 2015 Jun 1;10(6):e0128611. doi: 10.1371/journal.pone.0128611 (PMC4452488; doi:10.1371/journal.pone.0128611)
Supplement: S4 Table — (DOC) [file pone.0128611.s004.doc]

**S4 Table** Data for Fig 1 D: accumulated fermentation system mass loss of strain A.

| fermentation time (d) | accumulated mass loss (g) | | | |
| --- | --- | --- | --- | --- |
|  | 0 mM group | 0.5 mM group | 1 mM group | 1.5 mM group |
| 0 | 0 | 0 | 0 | 0 |
| 1 | 6.92±0.37 | 1.18±0.86 | 0.25±0.335 | 0.06±0.455 |
| 2 | 19.84±0.75 | 4.71±0.845 | 2.78±0.325 | 0.59±0.415 |
| 3 | 27±0.925 | 6.99±0.58 | 5.05±0.315 | 1.15±0.435 |
| 4 | 30.62±0.83 | 8.68±0.38 | 6.05±0.285 | 1.92±0.445 |
| 5 | 32.75±0.76 | 9.77±0.19 | 7.23±0.7 | 2.32±0.48 |
| 6 | 33.79±0.645 | 10.51±0.05 | 7.49±0.965 | 2.8±0.5 |
| 7 | 34.06±0.58 | 10.93±0.065 | 7.87±0.91 | 3.11±0.51 |
| 8 | 34.35±0.355 | 11.25±0.17 | 7.97±0.685 | 3.14±0.52 |
| 9 | 34.56±0.29 | 11.4±0.235 | 8.23±0.71 | 3.15±0.56 |
| 10 | 34.6±0.265 | 11.43±0.265 | 8.45±0.66 | 3.16±0.565 |
| 12 | 34.69±0.31 | 11.45±0.335 | 8.67±0.62 | 3.16±0.56 |
| 14 | 34.71±0.375 | 11.49±0.33 | 8.85±0.485 | 3.15±0.49 |
