# Supplementary material for: Copper Tolerance and Biosorption of Saccharomyces cerevisiae during Alcoholic Fermentation
Source: PLoS One. 2015 Jun 1;10(6):e0128611. doi: 10.1371/journal.pone.0128611 (PMC4452488; doi:10.1371/journal.pone.0128611)
Supplement: S5 Table — (DOC) [file pone.0128611.s005.doc]

**S5 Table** Data for Fig 1 E: accumulated fermentation system mass loss of strain B.

| fermentation time (d) | accumulated mass loss (g) | | | |
| --- | --- | --- | --- | --- |
|  | 0 mM group | 0.5 mM group | 1 mM group | 1.5 mM group |
| 0 | 0 | 0 | 0 | 0 |
| 1 | 5.93±0.41 | 0.73±0.135 | 0.27±0.845 | 0.14±0.335 |
| 2 | 17.97±0.36 | 2.07±0.145 | 1.07±0.58 | 0.73±0.325 |
| 3 | 24.67±0.315 | 6.09±0.16 | 3.99±0.38 | 2.53±0.315 |
| 4 | 29.4±0.29 | 8.35±0.17 | 5.67±0.19 | 3.31±0.305 |
| 5 | 32.71±0.25 | 9.66±0.175 | 7.14±0.05 | 4.03±0.305 |
| 6 | 34.02±0.23 | 10.21±0.185 | 8.39±0.065 | 4.67±0.31 |
| 7 | 34.16±0.235 | 10.89±0.23 | 8.89±0.17 | 5.03±0.375 |
| 8 | 34.24±0.18 | 11.68±0.225 | 9.26±0.235 | 5.26±0.385 |
| 9 | 34.37±0.17 | 11.79±0.22 | 9.32±0.265 | 5.35±0.385 |
| 10 | 34.43±0.135 | 11.83±0.225 | 9.34±0.335 | 5.37±0.253 |
| 12 | 34.51±0.125 | 11.89±0.225 | 9.35±0.33 | 5.38±0.236 |
| 14 | 34.51±0.115 | 12.03±0.225 | 9.35±0.345 | 5.38±0.221 |
