# Supplementary material for: Copper Tolerance and Biosorption of Saccharomyces cerevisiae during Alcoholic Fermentation
Source: PLoS One. 2015 Jun 1;10(6):e0128611. doi: 10.1371/journal.pone.0128611 (PMC4452488; doi:10.1371/journal.pone.0128611)
Supplement: S6 Table — (DOC) [file pone.0128611.s006.doc]

**S6 Table** Data for Fig 1 F: accumulated fermentation system mass loss of strain F.

| fermentation time (d) | accumulated mass loss (g) | | | |
| --- | --- | --- | --- | --- |
|  | 0 mM group | 0.5 mM group | 1 mM group | 1.5 mM group |
| 0 | 0 | 0 | 0 | 0 |
| 1 | 6.54±0.325 | 1.28±0.36 | 0.52±0.145 | 0.35±0.58 |
| 2 | 18.5±0.315 | 4.49±0.315 | 1.26±0.16 | 0.82±0.38 |
| 3 | 26.41±0.305 | 7.36±0.29 | 2.94±0.17 | 1.86±0.19 |
| 4 | 31.51±0.305 | 9.79±0.25 | 5.47±0.175 | 4.02±0.05 |
| 5 | 34.29±0.31 | 11.46±0.23 | 7.42±0.185 | 6.04±0.065 |
| 6 | 34.93±0.375 | 12.6±0.235 | 8.72±0.23 | 7.2±0.17 |
| 7 | 35.09±0.385 | 13.44±0.18 | 9.56±0.225 | 7.55±0.235 |
| 8 | 35.2±0.385 | 14.09±0.17 | 10.07±0.22 | 7.79±0.265 |
| 9 | 35.26±0.253 | 14.52±0.135 | 10.48±0.225 | 7.82±0.335 |
| 10 | 35.28±0.236 | 14.73±0.125 | 10.88±0.225 | 7.82±0.33 |
| 12 | 35.29±0.221 | 14.82±0.115 | 10.93±0.225 | 7.84±0.345 |
| 14 | 35.28±0.221 | 14.87±0.115 | 10.93±0.225 | 7.85±0.345 |
