# Supplementary material for: Copper Tolerance and Biosorption of Saccharomyces cerevisiae during Alcoholic Fermentation
Source: PLoS One. 2015 Jun 1;10(6):e0128611. doi: 10.1371/journal.pone.0128611 (PMC4452488; doi:10.1371/journal.pone.0128611)
Supplement: S7 Table — (DOC) [file pone.0128611.s007.doc]

**S7** **Table** Data for Fig 2 A: fermentation must reducing sugar of strain A.

| fermentation time (d) | reducing sugar (g/L) | | | |
| --- | --- | --- | --- | --- |
|  | 0 mM group | 0.5 mM group | 1 mM group | 1.5 mM group |
| 0 | 198.5456±1.58689 | 199.0458±0.89586 | 199.892±0.05895 | 200.238±0.225 |
| 1 | 158.26±0.9825 | 189.256±1.86282 | 194.856±0.25625 | 198.256±2.0458 |
| 2 | 105.58±3.5895 | 174.332±1.25625 | 183.565±1.5863 | 193.256±0.2354 |
| 4 | 26.58±0.58312 | 160.25±3.5891 | 173.568±0.2635 | 190.256±4.5689 |
| 6 | 9.586±0.00589 | 150.256±0.11256 | 165.658±0.88931 | 186.583±0.3954 |
| 8 | 4.158±0.1795 | 144.258±0.002892 | 159.256±0.1245 | 184.265±0.712 |
| 10 | 4.025±0.86586 | 138.256±0.17256 | 153.256±2.0589 | 183.256±3.2589 |
| 12 | 3.992±0.11578 | 135.256±0.8921 | 150.256±0.0823 | 182.985±0.15 |
| 14 | 3.956±0.22568 | 133.485±0.05831 | 149.398±0.678 | 182.264±0.823 |
