# Supplementary material for: Copper Tolerance and Biosorption of Saccharomyces cerevisiae during Alcoholic Fermentation
Source: PLoS One. 2015 Jun 1;10(6):e0128611. doi: 10.1371/journal.pone.0128611 (PMC4452488; doi:10.1371/journal.pone.0128611)
Supplement: S8 Table — (DOC) [file pone.0128611.s008.doc]

**S8 Table** Data for Fig 2 B: fermentation must reducing sugar of strain B.

| fermentation time (d) | reducing sugar (g/L) | | | |
| --- | --- | --- | --- | --- |
|  | 0 mM group | 0.5 mM group | 1 mM group | 1.5 mM group |
| 0 | 200.256±0.6826 | 198.568±0.1258 | 198.124±0.05895 | 199.586±0.1258 |
| 1 | 156.258±0.2568 | 186.586±0.3985 | 192.358±0.25625 | 197.582±0.2231 |
| 2 | 102.156±2.5698 | 170.256±2.589 | 180.256±2.856 | 190.256±0.3125 |
| 4 | 23.561±0.58312 | 155.235±4.25 | 171.258±0.45 | 183.258±0.264 |
| 6 | 8.953±0.069 | 148.256±0.69 | 162.358±3.256 | 175.682±5.328 |
| 8 | 4.123±0.3589 | 140.235±0.891 | 156.325±0.125 | 173.256±0.126 |
| 10 | 4.025±0.1587 | 133.258±0.263 | 151.325±0.6945 | 171.268±0.856 |
| 12 | 3.958±0.369 | 130.258±0.286 | 146.283±0.15 | 170.258±0.951 |
| 14 | 3.948±0.125 | 128.56±0.145 | 144.56±0.8411 | 169.85±0.126 |
