# Supplementary material for: Copper Tolerance and Biosorption of Saccharomyces cerevisiae during Alcoholic Fermentation
Source: PLoS One. 2015 Jun 1;10(6):e0128611. doi: 10.1371/journal.pone.0128611 (PMC4452488; doi:10.1371/journal.pone.0128611)
Supplement: S9 Table — (DOC) [file pone.0128611.s009.doc]

**S9 Table** Data for Fig 2 C: fermentation must reducing sugar of strain F.

| fermentation time (d) | reducing sugar (g/L) | | | |
| --- | --- | --- | --- | --- |
|  | 0 mM group | 0.5 mM group | 1 mM group | 1.5 mM group |
| 0 | 196.258±0.25948 | 198.364±0.5495 | 199.258±1.209 | 199.856±0.4591 |
| 1 | 152.354±0.648 | 183.256±0.98491 | 189.568±0.94156 | 193.586±0.1651 |
| 2 | 98.256±0.594 | 165.259±0.95165 | 175.356±0.149 | 182.258±1.549 |
| 4 | 21.586±0.8942 | 146.325±2.3159 | 163.258±4.159 | 173.586±2.849 |
| 6 | 7.562±0.12654 | 132.568±0.19216 | 153.586±0.941 | 165.893±0.10651 |
| 8 | 3.925±1.4156 | 125.345±0.1561 | 146.586±0.125 | 160.258±0.4159 |
| 10 | 3.92±0.48941 | 120.586±0.1981 | 141.258±0.6945 | 158.126±0.8941 |
| 12 | 3.918±0.6151 | 117.256±0.159 | 137.568±0.9849 | 157.68±0.156 |
| 14 | 3.92±0.849 | 116.358±0.849 | 135.368±0.126 | 157.35±0.1657 |
