# Supplementary material for: Copper Tolerance and Biosorption of Saccharomyces cerevisiae during Alcoholic Fermentation
Source: PLoS One. 2015 Jun 1;10(6):e0128611. doi: 10.1371/journal.pone.0128611 (PMC4452488; doi:10.1371/journal.pone.0128611)
Supplement: S10 Table — (DOC) [file pone.0128611.s010.doc]

**S10 Table** Data for Fig 2 D: fermentation ethanol concentration of strain A.

| fermentation time (d) | ethanol concentration (%) | | | |
| --- | --- | --- | --- | --- |
|  | 0 mM group | 0.5 mM group | 1 mM group | 1.5 mM group |
| 0 | 0 | 0 | 0 | 0 |
| 1 | 1.852±0.0085 | 0.81±0.0859 | 0.43±0.00549 | 0.18±0.0059 |
| 2 | 5.158±0.3159 | 1.26±0.0958 | 1.03±0.001987 | 0.39±0.06954 |
| 4 | 8.369±0.08213 | 1.96±0.1261 | 1.46±0.129 | 0.58±0.0984 |
| 6 | 10.258±0.09156 | 2.66±0.03165 | 2.09±0.126 | 0.82±0.159 |
| 8 | 10.9526±0.284 | 3.08±0.0051 | 2.46±0.09156 | 0.93±0.1589 |
| 10 | 10.96852±0.061495 | 3.38±0.09126 | 2.68±0.21594 | 0.95±0.09459 |
| 12 | 10.9826±0.054165 | 3.55±0.0984 | 2.73±0.05645 | 0.98±0.0591 |
| 14 | 11.01±0.0561 | 3.6±0.2645 | 2.78±0.08456 | 1.02±0.168489 |
