# Supplementary material for: Copper Tolerance and Biosorption of Saccharomyces cerevisiae during Alcoholic Fermentation
Source: PLoS One. 2015 Jun 1;10(6):e0128611. doi: 10.1371/journal.pone.0128611 (PMC4452488; doi:10.1371/journal.pone.0128611)
Supplement: S11 Table — (DOC) [file pone.0128611.s011.doc]

**S11 Table** Data for Fig 2 E: fermentation ethanol concentration of strain B.

| fermentation time (d) | ethanol concentration (%) | | | |
| --- | --- | --- | --- | --- |
|  | 0 mM group | 0.5 mM group | 1 mM group | 1.5 mM group |
| 0 | 0 | 0 | 0 | 0 |
| 1 | 2.085±0.0561 | 0.82±0.0859 | 0.46±0.00694 | 0.21±0.001459 |
| 2 | 5.536±0.0626 | 1.31±0.01465 | 1.21±0.0981 | 0.49±0.098489 |
| 4 | 8.485±0.0816 | 1.91±0.1268 | 1.53±0.0815 | 0.62±0.0612 |
| 6 | 10.368±0.1026 | 2.64±0.0984 | 2.26±0.006841 | 0.93±0.008419 |
| 8 | 10.869±0.00846 | 3.21±0.2159 | 2.78±0.0781 | 1.26±0.001859 |
| 10 | 10.968±0.0681 | 3.69±0.0984 | 2.89±0.2345 | 1.46±0.0945 |
| 12 | 11.01±0.0951 | 3.8±0.00894 | 2.98±0.00816 | 1.63±0.084 |
| 14 | 11.05±0.0984 | 3.85±0.0366 | 3.021±0.062 | 1.67±0.121 |
