# Supplementary material for: Copper Tolerance and Biosorption of Saccharomyces cerevisiae during Alcoholic Fermentation
Source: PLoS One. 2015 Jun 1;10(6):e0128611. doi: 10.1371/journal.pone.0128611 (PMC4452488; doi:10.1371/journal.pone.0128611)
Supplement: S12 Table — (DOC) [file pone.0128611.s012.doc]

**S12** **Table** Data for Fig 2 F: fermentation ethanol concentration of strain F.

| fermentation time (d) | ethanol concentration (%) | | | |
| --- | --- | --- | --- | --- |
|  | 0 mM group | 0.5 mM group | 1 mM group | 1.5 mM group |
| 0 | 0 | 0 | 0 | 0 |
| 1 | 2.12±0.0989 | 0.88±0.0649 | 0.6±0.0789 | 0.36±0.01264 |
| 2 | 5.69±0.08416 | 1.56±0.0165 | 1.22±0.0489 | 0.83±0.0984 |
| 4 | 8.69±0.0216 | 2.35±0.0894 | 1.93±0.10126 | 1.28±0.126 |
| 6 | 10.43±0.126 | 3.16±0.0481 | 2.79±0.00984 | 1.52±0.0489 |
| 8 | 10.96±0.0949 | 3.86±0.0651 | 3.16±0.0651 | 1.88±0.0984 |
| 10 | 10.99±0.01296 | 4.26±0.129 | 3.39±0.005948 | 2.06±0.13549 |
| 12 | 11.03±0.0894 | 4.51±0.0489 | 3.52±0.00159 | 2.31±0.0894 |
| 14 | 11.06±0.00894 | 4.56±0.008948 | 3.58±0.0654 | 2.38±0.008459 |
