# Supplementary material for: Copper Tolerance and Biosorption of Saccharomyces cerevisiae during Alcoholic Fermentation
Source: PLoS One. 2015 Jun 1;10(6):e0128611. doi: 10.1371/journal.pone.0128611 (PMC4452488; doi:10.1371/journal.pone.0128611)
Supplement: S13 Table — (DOC) [file pone.0128611.s013.doc]

**S13 Table** Data for Fig 3 A: copper ion concentration of MSM during fermentation for control group

| fermentation time (d) | copper concentration (mg/L) | | |
| --- | --- | --- | --- |
|  | 0.5 mM group | 1 mM group | 1.5 mM group |
| 0 | 31.9563±0.964 | 62.2158±0.8145 | 96.2875±0.0258 |
| 1 | 31.3145±0.1785 | 61.4895±1.1524 | 95.586±0.1235 |
| 2 | 31.0258±0.8561 | 61.0785±0.125 | 94.8562±0.2478 |
| 3 | 30.9325±1.258 | 60.8523±0.4885 | 94.3258±0.0878 |
| 4 | 30.7961±0.925 | 60.5842±1.0568 | 94.0831±0.05895 |
| 5 | 30.7258±0.5861 | 60.3148±0.2175 | 93.8215±0.03547 |
| 6 | 30.7058±0.5892 | 60.2895±0.6589 | 93.5847±0.02447 |
| 7 | 30.6952±0.3984 | 60.1987±0.0985 | 93.2475±0.0821 |
| 8 | 30.6825±1.1251 | 60.0657±0.1425 | 93.1058±0.00258 |
| 9 | 30.6842±0.8569 | 59.9275±0.9852 | 92.8452±0.00478 |
| 10 | 30.6838±0.2478 | 59.9856±0.5698 | 92.8017±0.089 |
| 12 | 30.6819±0.2588 | 59.9098±1.2285 | 92.7482±0.14 |
| 14 | 30.6828±0.0036 | 59.8762±0.2189 | 92.7852±0.0047 |
