# Supplementary material for: Copper Tolerance and Biosorption of Saccharomyces cerevisiae during Alcoholic Fermentation
Source: PLoS One. 2015 Jun 1;10(6):e0128611. doi: 10.1371/journal.pone.0128611 (PMC4452488; doi:10.1371/journal.pone.0128611)
Supplement: S14 Table — (DOC) [file pone.0128611.s014.doc]

**S14** **Table** Data for Fig 3 B: copper ion concentration of MSM during fermentation for strain A

| fermentation time (d) | copper concentration (mg/L) | | |
| --- | --- | --- | --- |
|  | 0.5 mM group | 1 mM group | 1.5 mM group |
| 0 | 30.95625±0.85 | 61.48125±0.8958 | 96.25±0.289 |
| 1 | 27.2563±0.01 | 57.625±0.5586 | 87.1875±1.589 |
| 2 | 22.1813±0.489 | 54.91875±0.5895 | 84.625±0.25 |
| 3 | 19.7313±0.5892 | 51.8125±0.4885 | 82.5±0.058 |
| 4 | 16.375±0.1589 | 48.21875±1.0568 | 78.25±0.8958 |
| 5 | 14.9125±1.058 | 47.45±0.0058 | 76.125±0.27859 |
| 6 | 13.0154±0.5892 | 46.575±0.6589 | 75.125±0.058 |
| 7 | 12.3875±0.3984 | 44.875±0.0789 | 74.0625±1.85982 |
| 8 | 10.925±0.0258 | 44.36875±0.5895 | 72.4375±0.5865 |
| 9 | 10.2568±0.8569 | 43.96875±0.3658 | 71.375±0.03568 |
| 10 | 10.0125±0.2478 | 43.55±0.1184 | 70.1875±0.0425 |
| 12 | 9.9897±0.2588 | 42.49375±0.2225 | 69.125±0.5836 |
| 14 | 9.9874±0.0036 | 42.80625±0.2189 | 69.05±0.0785 |
