# Supplementary material for: Copper Tolerance and Biosorption of Saccharomyces cerevisiae during Alcoholic Fermentation
Source: PLoS One. 2015 Jun 1;10(6):e0128611. doi: 10.1371/journal.pone.0128611 (PMC4452488; doi:10.1371/journal.pone.0128611)
Supplement: S15 Table — (DOC) [file pone.0128611.s015.doc]

**S15 Table** Data for Fig 3 C: copper ion concentration of MSM during fermentation for strain B

| fermentation time (d) | copper concentration (mg/L) | | |
| --- | --- | --- | --- |
|  | 0.5 mM group | 1 mM group | 1.5 mM group |
| 0 | 32.125±0.058954 | 59.625±0.00589 | 93.875±0.5895 |
| 1 | 29.75±0.12589 | 58.075±0.5896 | 87.375±0.98658 |
| 2 | 25.1433±0.26895 | 55.6±0.9865 | 84.875±0.2589 |
| 3 | 21.9313±0.589875 | 50.19375±0.25894 | 79.6875±0.2589 |
| 4 | 19.0625±0.6985 | 48.25625±0.1256 | 77.3125±0.4789 |
| 5 | 17.0225±0.8956 | 47.7±0.23589 | 75.875±0.2578 |
| 6 | 15.4485±1.0258 | 45.49375±0.3 | 74.9375±0.6928 |
| 7 | 13.5821±0.22368 | 44.6375±0.598 | 75±1.2568 |
| 8 | 12.5685±0.2895 | 43.4125±0.9688 | 72.9375±0.2589 |
| 9 | 12.0895±0.004895 | 45.29375±0.4885 | 70.75±0.1478 |
| 10 | 11.9861±0.0589 | 42.44375±0.1568 | 72.5625±0.2816 |
| 12 | 11.8612±0.8964 | 42.08125±0.2568 | 72.375±0.1367 |
| 14 | 11.8489±0.7892 | 42.2±0.15978 | 69.75±0.8592 |
