# Supplementary material for: Copper Tolerance and Biosorption of Saccharomyces cerevisiae during Alcoholic Fermentation
Source: PLoS One. 2015 Jun 1;10(6):e0128611. doi: 10.1371/journal.pone.0128611 (PMC4452488; doi:10.1371/journal.pone.0128611)
Supplement: S16 Table — (DOC) [file pone.0128611.s016.doc]

**S16** **Table** Data for Fig 3 D: copper ion concentration of MSM during fermentation for strain F

| fermentation time (d) | copper concentration (mg/L) | | |
| --- | --- | --- | --- |
|  | 0.5 mM group | 1 mM group | 1.5 mM group |
| 0 | 29.73±0.2589 | 61.19±0.5897 | 91.63±0.3698 |
| 1 | 28.25±0.1898 | 59.49±0.9858 | 87.5±0.2658 |
| 2 | 25.66±0.5895 | 57.21±1.2569 | 85.44±3.549 |
| 3 | 22.25±0.8978 | 53.51±0.25 | 82±0.8698 |
| 4 | 18.87±0.257 | 51.24±0.0369 | 83.19±0.2584 |
| 5 | 18.4±0.6589 | 51.13±0.0098 | 79.44±0.00369 |
| 6 | 17.71±0.00859 | 49.08±0.247 | 78.56±0.36548 |
| 7 | 16.05±0.0458 | 48.8±0.3697 | 77.31±0.2156 |
| 8 | 14.95±0.15798 | 47.69±0.15 | 77.88±0.259 |
| 9 | 13.63±0.0879 | 47.39±0.0872 | 77.55±1.0326 |
| 10 | 13.43±0.4589 | 47.16±0.6985 | 77.81±0.2594 |
| 12 | 13.33±0.2258 | 47.89±0.1235 | 78.69±0.4895 |
| 14 | 13.28±0.267 | 47.03±0.3694 | 78.01±0.2354 |
