# Supplementary material for: Copper Tolerance and Biosorption of Saccharomyces cerevisiae during Alcoholic Fermentation
Source: PLoS One. 2015 Jun 1;10(6):e0128611. doi: 10.1371/journal.pone.0128611 (PMC4452488; doi:10.1371/journal.pone.0128611)
Supplement: S17 Table — (DOC) [file pone.0128611.s017.doc]

**S17 Table** Data for Fig 4 A: removal ratio ηof Cu^2+^ on *S. cerevisiae* strains AWRI R2 (A), BH8 (B) and Freddo (F) at the end of alcoholic fermentation in MSM with 0.50, 1.00 and 1.50 mM Cu^2+^.

|  | removal ratio η (%) | | |
| --- | --- | --- | --- |
|  | 0.5 mM group | 1 mM group | 1.5 mM group |
| Control | 3.9851±0.162 | 3.7604±0.324 | 3.6373±0.585 |
| AWRI R2 | 67.371±0.697 | 30.38±0.462 | 28.26±0.235 |
| BH8 | 63.1163±0.398 | 29.56±0.495 | 25.69±0.126 |
| Freddo | 55.33±0.495 | 23.14±0.346 | 14.86±0.427 |
