# Supplementary material for: Copper Tolerance and Biosorption of Saccharomyces cerevisiae during Alcoholic Fermentation
Source: PLoS One. 2015 Jun 1;10(6):e0128611. doi: 10.1371/journal.pone.0128611 (PMC4452488; doi:10.1371/journal.pone.0128611)
Supplement: S18 Table — (DOC) [file pone.0128611.s018.doc]

**S18 Table** Data for Fig 4 B: adsorption efficiency *A* of Cu^2+^ on *S. cerevisiae* strains AWRI R2 (A), BH8 (B) and Freddo (F) at the end of alcoholic fermentation in MSM with 0.50, 1.00 and 1.50 mM Cu^2+^.

|  | adsorption efficiency *A* (mg/g) | | |
| --- | --- | --- | --- |
|  | 0.5 mM group | 1 mM group | 1.5 mM group |
| AWRI R2 | 7.83±0.323 | 8.46±0.363 | 15.82±0.604 |
| BH8 | 7.58±0.49 | 7.84±0.258 | 12.31±0.307 |
| Freddo | 5.88±0.89 | 6.24±0.349 | 8.86±0.591 |
